# COVID-19 vaccination-related headache showed two different clusters in the long-term course: a prospective multicenter follow-up study (COVA-Head Study)

**DOI:** 10.1186/s10194-023-01665-3

**Published:** 2023-09-29

**Authors:** Arife Çimen Atalar, Ayşe Nur Özdağ Acarlı, Betül Baykan, Paolo Martelletti, Hayrunnisa Bolay, Mustafa Ertaş, Esme Ekizoğlu, Ömer Karadaş, Burcu Polat, Işıl Yazıcı Gençdal, David Garcia Azorin, Dimos Mitsikostas, Loukia Apostolakopoulou, Hamit Genç, Pınar Yalınay Dikmen, Esra Acıman Demirel, Elif Ilgaz Aydınlar, Rabia Gökçen Gözübatık-Celik, Javid Shafiyev, Bahar Taşdelen, Aynur Özge

**Affiliations:** 1 Department of Neurology, Health Sciences University, Kanuni Sultan Süleyman Education and Research Hospital, Istanbul, Turkey; 2 Department of Neurology, Ermenek State Hospital, Karaman, Turkey; 3 Department of Neurology, Headache Center, Istanbul University, Istanbul Faculty of Medicine, Istanbul, Turkey; 4 Department of Neurology, EMAR Medical Center, Istanbul, Turkey; 5 Department of Clinical and Molecular Medicine, Sapienza University, Rome, Italy; 6 Medical Faculty, Department of Neurology and Algology, Gazi University, Ankara, Turkey; 7 Department of Neurology, University of Health Sciences, Gulhane Training and Research Hospital, Ankara, Turkey; 8 School of Medicine, Department of Neurology, Istanbul Medipol University, Istanbul, Turkey; 9 Bakirkoy Prof. Dr. Mazhar Osman Training and Research Hospital for Psychiatric, Neurologic and Neurosurgical Diseases, University of Health Sciences, Istanbul, Turkey; 10 Department of Neurology, Hospital Clínico Universitario de Valladolid, Headache Unit, Valladolid, Spain; 11 First Neurology Department, Medical School, Aeginition Hospital, National and Kapodistrian University of Athens, Athens, Greece; 12 University of Health Sciences, Van Training and Research Hospital, Van, Türkiye; 13 School of Medicine, Department of Neurology, Acibadem Mehmet Ali Aydinlar University, Istanbul, Turkey; 14 Bülent Ecevit University Neurology Department, Zonguldak, Turkey; 15 Department of Biostatistics and Medical Informatic, Mersin University School of Medicine, Mersin, Turkey; 16 Medical Faculty, Department of Neurology, Mersin University, Mersin, Türkiye

**Keywords:** COVID, Vaccination, Long-COVID, Headache, Primary headache

## Abstract

**Background:**

Although acute headache following COVID-19 vaccination is widely acknowledged, the long-term progression of these headaches remains poorly understood. Our objective was to identify various phenotypes of prolonged or worsened headaches associated with COVID-19 vaccination and document any changes in these phenotypes over an extended period. Additionally, we aimed to document the diverse headache presentations among patients with pre-existing primary headaches.

**Methods:**

A multinational, prospective observational study was conducted to investigate prolonged or worsened headaches associated with COVID-19 vaccination. Questionnaires assessing COVID-19 vaccination-related headaches at three time points (initial visit, 3^rd^ month follow-up, and 6th month follow-up) were developed for the study. Headache specialists/clinicians evaluated patients using these questionnaires in a prospective manner. Repeated K-means cluster analysis was performed to identify patient profiles with prolonged or worsened headaches related to COVID-19 vaccination.

**Results:**

Among the 174 patients included in the study, there was a female-to-male ratio of 128 (73.6%) to 46 (26.4%). The mean age of the patient group was 45.2 ± 13.3 years, and 107 patients (61.5%) had a pre-existing history of primary headaches. Through the analysis, two major clusters were identified based on headache characteristics at each visit. During the first visit (*n* = 174), Cluster 1 primarily comprised patients with a history of primary headaches, frontal localization of pain, throbbing pain type, more severe headaches accompanied by symptoms such as nausea, phonophobia, photophobia, and osmophobia, and worsened by physical activity. In contrast, Cluster 2 consisted of patients with longer headache durations (over one month) and a stabbing/pressing quality of pain. Patients in Cluster 1 had a higher prevalence of migraine as the pre-existing primary headache disorder compared to Cluster 2 (90.48% vs. 68.18%, respectively; *p* = 0.005).

**Conclusion:**

The identification of two distinct phenotypes of prolonged or worsened headaches related to COVID-19 vaccination can provide valuable clinical insights. Having an awareness of the potential worsening of headaches following COVID-19 vaccination, particularly in patients with a primary headache disorder such as migraine, can help clinicians and headache experts anticipate and adjust their treatment strategies accordingly. This knowledge can aid in preplanning treatment modifications and optimize patient care.

**Supplementary Information:**

The online version contains supplementary material available at 10.1186/s10194-023-01665-3.

## Introduction

After the emergence of the COVID-19 pandemic, vaccination has become the most promising and effective weapon against the infection. Since then, vaccines against COVID-19 have played a major role in preventing the spread of the virus and reducing the disease-related mortality worldwide and still maintain their importance where there is a lack of certain effective medication or treatment [1]. As a result, the development of new vaccines specific to SARS-CoV-2 has accelerated globally [1]. Since early December 2020, several different vaccines have been approved by the European Medicines Agency (EMA). These include BNT162b2 (Comirnaty by Pfizer-BioNTech), an mRNA vaccine, followed by mRNA-1273 (Moderna), Oxford-AstraZeneca vaccine AZD1222 (ChAdOx1), JNJ-78436735 (Ad26.COV2.S by Janssen), along with 178 vaccines currently in the clinical development stage [2]. These vaccines have various adverse effects, with headache being one of the most frequently reported among the spectrum of neurological side effects [3–5]. The frequency, clinical characteristics, phenotypes, and pathophysiological basis of headaches following COVID-19 vaccination have become a new area of investigation with numerous unanswered questions [6]. These include the condition of patients with pre-existing headaches or a history of COVID-19, the long-term course of these headaches, patients in need of prophylactic medication against headaches before vaccination, and patients who require further diagnostic investigations.

Currently, there are a considerable number of papers focused on the acute presentations of COVID-19 vaccine-related headaches, revealing that headaches are a frequent and disabling problem in up to half of the vaccine recipients, with the majority of them experiencing symptoms consistent with tension-type headache [6–8]. Classically, headache occurs within the first 72 h after vaccination and is frequently accompanied by systemic symptoms such as fever, myalgia, diarrhea, arthralgia, and fatigue [7]. Although post-vaccination headache in the acute setting is now well-recognized, little is known about the long-term course of these headaches. Whether these headaches subside over time or persist as part of long-COVID symptoms are some of the unclear points that require clarification [9]. Currently, only a few papers have reported on the long-term course of COVID-19 vaccination-related headaches, with a focus on headache characteristics. However, the effects of a prior primary headache disorder on the course of vaccination-related headaches are still not fully understood [4].

In this multinational, multicenter, and prospective study, our aim was to identify different phenotypes of COVID-19 vaccination-related prolonged/worsened headaches and document the changes in these phenotypes over the long-term course. For this aim, we used a standardized questionnaire administered by headache experts during the first visit and two follow-up visits (in the 3^rd^ and 6^th^ months after vaccination). As a secondary aim, the questionnaire included detailed questions about patients' pre-vaccination and post-vaccination headache conditions, as well as accompanying long COVID symptoms.

## Methods

### Study design and patient selection

A multinational, prospective observational study focused on COVID-19 vaccination-related prolonged or worsened headaches was organized by a scientific committee composed of seven headache experts. After several online discussions, two committee members (AÇA, ME) developed three structured questionnaires on the Google Forms platform for neurologists/headache experts to enter the data of their patients with prolonged/worsened headaches following COVID-19 vaccination. Three time points were determined for patient evaluation: the first visit and two follow-up visits at the 3^rd^ and 6^th^ months. The expert committee reviewed and approved all study questionnaires.

These questionnaires included targeted questions about the demographics and baseline characteristics of patients with prolonged/worsened COVID-19 vaccination-related headaches. The questionnaires explored the time relationship between headache and vaccination, frequency, duration, localization, quality, lateralization, severity, accompanying symptoms, treatment choices, possible secondary etiologies, and long COVID symptoms. Additionally, the questionnaires included detailed inquiries about the patients' previous COVID-19 history and any pre-existing primary headache diagnosis. The severity of headaches was determined using a 4-point verbal rating scale (VRS), which is widely employed for evaluating pain intensity due to its simplicity, understandability, and adaptability. This scale is recommended by the International Headache Society (IHS) guidelines for use in controlled trials [10]. Patient responses are graded as follows: (0) No pain, (1) mild (non-irritating and not interfering with daily work), (2) moderate (uncomfortable but still able to perform daily work), and (3) severe (unable to perform daily work).

We also incorporated the Mig-SCog scale to measure and monitor cognitive symptoms associated with COVID-19 vaccination-related headaches during the first and follow-up visits. The Mig-SCog scale is a specific instrument designed to assess subjective cognitive symptoms during migraine attacks. It is a Likert-type scale consisting of 9 items scored between 0 and 18. Questions 1–3 pertain to attention, processing speed, and orientation; questions 4–5 relate to planning and attention; questions 6–7 assess language; and questions 8–9 focus on language naming. The Mig-SCog scale aims to investigate the areas that cause the most complaints in patients during an attack, specifically executive functions (attention, planning, and orientation) and language (naming and language comprehension). Scoring is as follows: often (2 points), sometimes (1 point), and none (0 points). The total score is the sum of these individual scores. A higher Mig-SCog score indicates a higher frequency of cognitive symptoms. This scale is widely used and recognized as a valid and reliable instrument in many countries worldwide [11].

The follow-up questionnaires at the 3^rd^ and 6^th^ months included additional inquiries regarding the course of vaccination-related prolonged/worsened headaches and the course of long-COVID-19 symptoms (Supplementary files 1–3). The diagnosis of patients with pre-existing primary headaches was also reassessed and confirmed in accordance with the International Classification of Headache Disorders (ICHD)-3 criteria [12].

### Data collection

Initially, neurologists/headache experts from Asia, Europe, and the USA were invited via private emails, introducing the fundamental concepts of the project. Those experts who expressed their willingness to contribute to the study were provided with further information about the project's details, and online links to access the questionnaires were sent to them. Eventually, a total of twenty investigators from four countries and twelve headache centers agreed to participate in the study. Data collection was carried out prospectively from December 2021 to January 2023, commencing immediately after obtaining approval from the ethics committee. The study coordinator (AÇA) and the local coordinator (AÖA) obtained ethics committee approval from the Clinical Research Ethics Committee of the University of Health Sciences, Istanbul Education and Research Hospital (5.11.2021/2976). Subsequently, they conducted individual online visits to all centers to discuss the study protocol and organize follow-up visits accordingly. Furthermore, each participating center obtained their own ethical consent before contributing to the study.

As a prerequisite for participation, the onset of headache in the included patients should occur within the first 15 days after any type of COVID-19 vaccination. This requirement aims to document a clear temporal relationship between the headache and the vaccination, which is supported by patient files or headache diaries. Furthermore, the headache should start within the time window of 0–72 h after COVID-19 vaccination and extend beyond this period for inclusion, lasting longer than 14 days. Other inclusion criteria were as follows:

-Age between 18 and 65 years.
-Patients with or without pre-existing headaches could be included, but patients with a pre-existing primary headache should have been followed up for at least one year by the same headache center. If not, they should have either complete documentation of their medical records or a well-documented headache diary.
-Patients should have undergone a minimum of one brain neuroimaging [magnetic resonance imaging (MRI) or computerized tomography (CT)] and at least one routine blood biochemistry and hemogram.

We excluded patients with acute COVID-19 vaccination-related headaches who lacked complete documentation of their medical records or headache diaries regarding their previous primary headaches. Additionally, patients with mental disabilities that would hinder their participation in the study were also excluded.

The questionnaires were administered by the physicians/neurologists who were responsible for the patients' treatment. The initial visit included face-to-face interviews, and for the third and sixth months after the first visit, the interviews were conducted either face-to-face or by telephone.

Informed consent was obtained from each participant before their inclusion in the study.

**Fig. 1:**
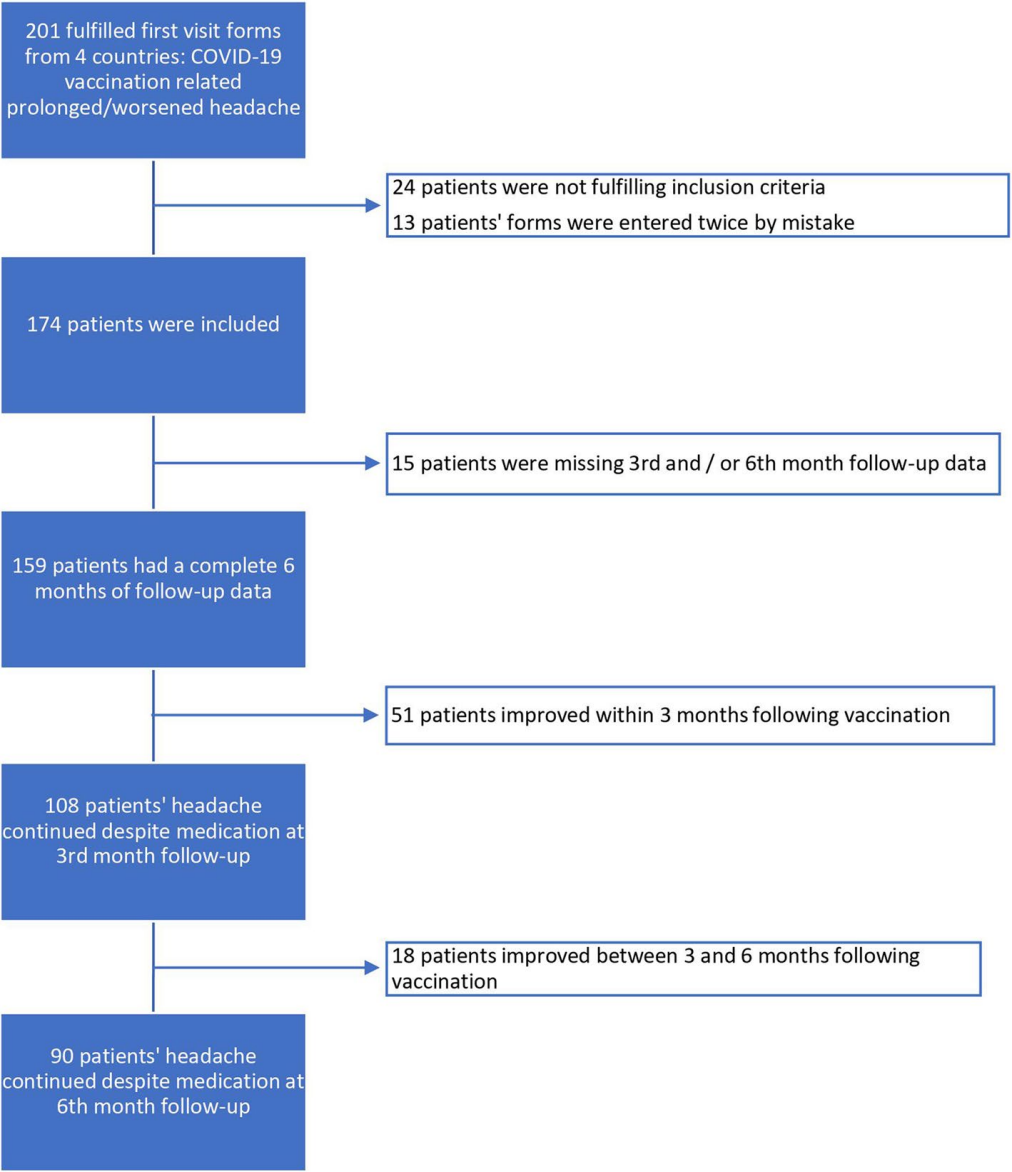
Flow chart of the study

Figure 1 provides a flowchart summarizing the study's process.

### Statistical analysis

Prior to patient recruitment, a power analysis was performed to determine the minimum number of patients required for the study. The calculation, conducted using the Epi Info software, resulted in a minimum recruitment target of 115 patients, with 80% power and 5% type I error level.

Categorical data were summarized as counts with percentages, while continuous data were presented as mean ± standard deviation (if assuming normality) or as median (min–max) values. Associations between categorical variables were assessed using Chi-square or Exact tests. Independent groups t-test or Mann Whitney U test were employed to compare groups with continuous variables.

To identify patient profiles with COVID-19 vaccination-related prolonged or worsened headaches, repeated K-means cluster analysis was conducted. Initially, clusters were estimated for 174 patients with headaches, and subsequent cluster analyses were performed for patients with continued headaches during the 2^nd^ (*n* = 106) and 3^rd^ (*n* = 82) visits. Factors influencing clustering were determined using chi-square tests and ANOVA within cluster analysis. The concordance of patient visits within clusters was analyzed using the McNemar test, and the rate of patients accumulating in the same cluster in the current visit was compared with the previous visit in terms of cluster accumulation. Changes in mean pain severity, number of headache days, and Mig-SCog scale throughout patient visits were analyzed using ANOVA and Friedman tests.

All statistical analyses were conducted using STATISTICA 13.0 [13]. A *p*-value of less than 0.05 was considered statistically significant.

## Results

A total of 174 patients were included in the study. During the 6-month follow-up period, 159 patients successfully completed all three follow-up visits (initial visit, 3^rd^ month, and 6^th^ month control visits), as depicted in the flow chart provided.

### The findings in the first visit

Among the patients, there were 128 females (73.6%) and 46 males (26.4%), resulting in a predominantly female population. The average age of the patient group was 45.2 ± 13.3 years. Within the main group, 107 patients (61.5%) had a pre-existing history of primary headaches, while 116 patients (66.7%) had additional systemic comorbidities, such as sleep problems, hypertension, anxiety disorder, asthma, and others.

Forty-six percent (80 patients) of the total study population had a history of COVID-19, while the remaining 54% (94 patients) did not experience COVID-19 at the time of the first visit. Among the 80 patients with a COVID-19 history, 51 of them (63.8%) reported experiencing headaches related to the infection.

Table 1 presents the demographic characteristics of patients with prolonged COVID-19 vaccination-related headaches at the first visit.

**Table 1:** The characteristics of the total patient group

**Demographic features (*n* = 174)**	
Sex (F/M) (n, %)	128/46 (73.6/26.4)
Age, mean ± SD, min–max	45.2 ± 13.3 (18–81)
Mig-SCog, mean ± SD	5.73 ± 4.90
Patients with a comorbid systemic disease (n, %)	116 (66.7)
**Pre-existing headache history (n, %)**	**107 (61.5)**
-Pre-existing headache types (n, %)	
–Migraine	85 (79.4)
–Tension-type headache	13 (12.1)
–Trigeminal autonomic headache	2 (1.9)
–Migraine + Medication overuse headache	2 (1.9)
–Other primary headache types	5 (4.7)
COVID-19 history at the referral (n, %)	80 (46)
Presence of headache in patients with COVID-19 history (n, %)	51 (63.7)
**Other long COVID symptoms (among patients with COVID-19 history) (n, %)**	
-Fatigue	55 (68.8)
-Cognitive involvement	36 (45)
-Psychiatric symptoms	9 (11.3)
-Sleep disorders	22 (27.5)
-Hair loss	23 (29.1)
**Secondary headache diagnosis related to COVID-19 vaccination**	8 (4.6)

*Abbreviations:* *F* female, *M* Male, *n* number, *SD* Standard deviation, *%* percentage

Table 2 presents the clinical characteristics of patients with prolonged/worsened COVID-19 vaccination-related headaches, specifically focusing on the comparison between patients with pre-existing primary headaches and those without primary headaches.

**Table 2:** Clinical characteristics of headache in patients with prolonged COVID-19 vaccine related headaches in relation to preexisting primary headache

Clinical/ demographic properties	Pre-existing PH ( +) (*n* = 107)	No pre-existing PH (*n* = 67)	*p*
Age, (mean ± SD)	46.03 ± 13.06	43.8 ± 13.8	0.228
Gender (f/m), (n/%)	88/19 (82.2/17.8)	40/27 (59.7/40.3)	**0.001**
Comorbid systemic disease, n (%)	75 (70.1)	41 (61.2)	0.226
COVID-19 related headache history ( +), n (%)	32 (61.5)	19 (67.9)	0.575
***COVID-19 vaccination related prolonged headache characteristics***			
Time interval between headache and vaccination (days), (mean ± SD)	2.77 ± 3.6	3.55 ± 3.57	**0.005**
Duration of headache attack ≥ 24 h, n (%)	86 (86.4)	57 (85.1)	0.430
Duration of headache ˃1 month, n (%)	39 (36.4)	41 (61.2)	**0.001**
Lateralization (unilateral/bilateral), n (%)	45/62 (42.1/57.9)	21/46 (31.3/68.7)	0.113
Quality, n (%)			
Throbbing	64 (59.8)	29 (43.3)	**0.033**
Pressing	49 (45.8)	47 (70.1)	**0.002**
Stabbing	15 (14)	13 (19.4)	0.347
Other	7 (6.5)	6 (9)	0.556
Localization, n (%)			
Frontal	62 (57.9)	32 (47.8)	0.190
Vertex	15 (14)	18 (26.9)	**0.035**
Temporal	42 (39.3)	22 (32.8)	0.393
Occipital	35 (32.7)	19 (28.4)	0.546
Holocranial	23 (21.5)	12 (17.9)	0.566
Other	7 (6.5)	4 (6)	0.880
Accompanying symptoms, n (%)			
Nausea	54 (50.5)	19 (28.4)	**0.004**
Phonophobia	55 (51.4)	25 (37.3)	**0.07**
Photophobia	58 (54.2)	23 (34.3)	**0.011**
Osmophobia	21 (19.6)	5 (7.5)	**0.029**
Dizziness	26 (24.3)	22 (32.8)	0.22
Increase by physical activity	44 (41.1)	24 (35.8)	0.486
Cranial autonomic symptoms	10 (9.3)	6 (9)	0.931
Allodynia	28 (26.2)	16 (23.9)	0.735
Other	25 (23.4)	11 (16.4)	0.271
Headache improvement status, n (%)			
Improved without medication	10 (9.3)	8 (11.9)	0.103
Totally relieved with medication	43 (40.2)	23 (34.3)	
Continued despite medication (no emergency referral)	47 (43.9)	24 (35.8)	
Emergency room/hospital referral due to headache	7(6.5)	12 (17.9)	
Other secondary headache diagnosis n (%)	1 (0.9)	7 (10.4)	**0.017**
Mig-SCog Scale (mean±SD)	6.56 ± 5.06	5.19 ± 4.82	0.076
Treatment choices n (%)			
Paracetamol	61 (57)	42 (62.7)	0.458
NSAID	41 (38.3)	34 (50.7)	0.107
Aspirin	11 (10.3)	6 (9)	0.775
Triptans	26 (24.3)	3 (4.5)	**0.001**
Ergotamine	3 (2.8)	3 (4.5)	0.556
Beta blockers	6 (5.6)	2 (3)	0.422
TCADs	8 (7.5)	6 (9)	0.727
SSRI	10 (9.3)	5 (7.5)	0.667
SNRI	1 (0.9)	5 (7.5)	**0.022**
Steroids	4 (3.7)	4 (6)	0.494
Muscle relaxants	3 (2.8)	9 (13.4)	**0.007**
GON block	13 (12.1)	9 (13.4)	0.804
Botulinum toxin	7 (6.5)	0 (0)	**0.033**
CGRP receptor antagonists	9 (8.4)	2 (3)	0.152

*Abbreviations:* *CGRP* Calcitonin gene related peptide, *f* female, *GON* Great occipital nerve, *m* male, *n* number, *PH* Primary headache, *SD* Standard deviation, *SNRI* Serotonin noradrenaline reuptake inhibitor, *SSRI* Selective serotonin reuptake inhibitors, *TCADs* Tricyclic antidepressants

Among all patients experiencing COVID-19 vaccination-related headaches, 8 (4.6%) patients were diagnosed with other secondary headaches. The specific secondary headache diagnoses were as follows: cerebral venous thrombosis (5 cases, 62.5%), intracranial hypertension (1 case, 12.5%), dental pain (1 case, 12.5%), and chronic sinusitis (1 case, 12.5%). One patient exhibited an increased platelet count, while another had elevated D-dimer levels. Cerebrospinal fluid analysis within normal limits was observed in 3 patients. Additionally, among the 8 patients with secondary headache diagnoses, 5 reported fatigue, 3 experienced muscle pain, 3 had joint pain, 2 had mild fever, and 1 had itching complaints.

### The findings during the follow-up

**Fig. 2:**
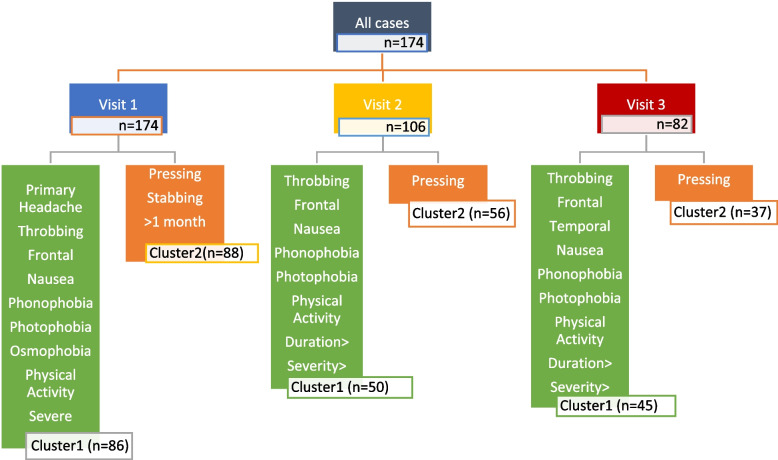
The results of the K repeated cluster analysis of COVID-vaccination related headache Abbreviations: n, number

In order to evaluate the long-term changes in COVID-vaccination related headache characteristics, we employed a repeated K cluster analysis method. All 174 patients were included in the cluster analysis at the 1^st^ visit (Fig. 2). However, at the 3^rd^ month, only 108 and at the 6^th^ month 90 patients still had ongoing headaches. Since 2 patients at the 3^rd^ month and 9 patients at the 6^th^ month had missing data, we could have included 106 patients from the 3^rd^ and 82 patients from the 6^th^ month for K-repeated analysis.

During each patient visit (baseline, 3^rd^ month, and 6^th^ month), two major clusters were identified based on headache characteristics. In the first visit (baseline, n = 174), Cluster 1 consisted of patients with throbbing, more severe headaches accompanied by symptoms such as nausea, phonophobia, photophobia, and osmophobia. These headaches were often exacerbated by physical activity. The majority of patients in this cluster had a history of primary headaches, and the pain was frequently localized in the frontal area.

Cluster 2, on the other hand, included patients with longer headache duration (greater than one month) along with stabbing or pressing quality of pain.

It was found that in Cluster 1, the prevalence of migraine as the pre-existing primary headache disorder was significantly higher (90.48% vs 68.18% respectively (*p* = 0,005)) compared to Cluster 2. On the other hand, tension-type headache history was more prevalent in Cluster 2 (22.73% vs 4.76%).

These clusters persisted during the subsequent follow-up visits with minor changes, notably. In the 3^rd^ month visit (*n* = 106), cluster 1 exhibited the same headache characteristics as in the 1^st^ visit, but with the addition of patients experiencing a longer duration of headache who were now included in this cluster. In contrast, cluster 2 consisted solely of patients with pressing headache. Finally, at the 6th month follow-up, clusters 1 and 2 remained the same as in the 3^rd^ month visit, except that patients with temporal location of headache were also included in the first cluster (Supplementary Table 4 a, b and c).

When we conducted concordance analysis between patient visits, the results showed that 71.42% of the patients grouped in Cluster 1 and 86.04% of the patients grouped in Cluster 2 at the 2^nd^ visit were placed in the same clusters as in the 1^st^ visit, indicating a concordance between the first two visits (McNemar *p* = 0.439).

Similarly, the 2^nd^ and 3^rd^ visits also showed concordance (McNemar *p* = 0.15441). The majority (93.33%) of the patients grouped in Cluster 1, and 78.38% of the patients in Cluster 2 at the 3^rd^ visit were placed in the same clusters as in the 2^nd^ visit.

**Fig. 3:**
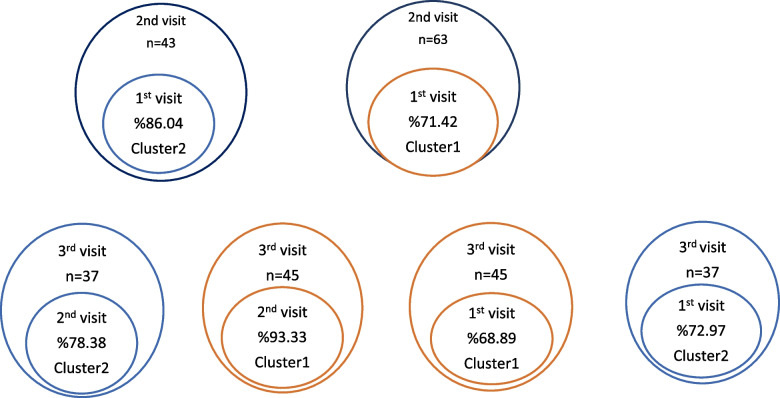
The graphical demonstration of the concordance between the visits of the patients in the longitudinal cluster analysis

Lastly, there was concordance between the first visit and the 3^rd^ visit (6^th^ month visit) of the patients (McNemar *p* = 0.69364). Figure 3 presents a graphical representation of the concordance between the visits of patients in terms of longitudinal cluster analysis.

At the end of the 6-month follow-up period, 45 out of 86 patients (52.32%) in Cluster 1 and 37 out of 88 patients (42.04%) in Cluster 2 still experienced ongoing (prolonged) headaches.

Furthermore, we conducted an analysis of the longitudinal changes in mean headache severity between the initial visit and follow-up visits using the Friedman ANOVA test. The results showed a significant decrease in headache severity during the follow-up period (2.48 ± 0.61, 2.19 ± 0.66, and 2.07 ± 0.72, respectively) (*p* < 0.0001). The comparison of Mig-SCog scale scores between patient visits revealed a gradual decrease in scores with each subsequent visit (5.73 ± 4.90, 4.48 ± 4.77, and 3.98 ± 4.73, respectively; *p* < 0.0001).

## Discussion

To the best of our knowledge, this study is the first multinational, prospective, and long-term follow-up investigation that sheds light on the phenotypic differences of patients with COVID-19 vaccination-related prolonged/worsened headaches. Our findings reveal that these headaches can be categorized into two distinct clusters based on their headache phenotypes. Cluster 1 comprises patients with migraine-like headache features, characterized by throbbing, more severe headaches primarily localized in the frontal region. These patients have a positive history of primary headaches and experience accompanying symptoms such as nausea, photo/phono/osmophobia, and worsened symptoms with physical activity. In contrast, patients in Cluster 2 present with pressing or stabbing headaches of longer duration (more than 1 month). An additional noteworthy finding of our study is the persistence of headache characteristics observed in patients within these Clusters during the long-term (6-month) prospective follow-up period. Although there was a trend of improvement in headache symptoms over time, the overall phenotypic profile of these patients remained relatively stable. This indicates that the specific headache phenotypes identified in the Clusters maintained their characteristics and did not undergo significant changes throughout the follow-up period.

### Characteristics of COVID-19 vaccination related headaches, and headache features in patients with a primary headache disorder history

Headache was reported as the fifth most common adverse event following COVID-19 vaccination during the acute phase [14]. Most observations report that headache typically starts within a time window of 72 h after the vaccination process [3, 6, 15]. In a recent study that evaluated new or worsening headaches within 16 days after COVID-19 vaccination, it was found that 289 patients experienced headaches that were temporally correlated with the vaccination. The study reported that regardless of the classification of headache (primary vs. secondary) or the type of vaccine, a migraine-like clinical phenotype was observed in these patients [16]. In a study by Ekizoğlu et al., the authors evaluated vaccine-related headache characteristics in 1819 healthcare personnel who predominantly received the inactivated vaccine (CoronaVac; Sinovac Life Sciences). The study found that headache was more predominant in the female population, with a higher prevalence compared to males (36.1% vs. 19.3%). Additionally, a subset of participants (12.1–16.9%) reported accompanying symptoms resembling migraine, such as nausea, photophobia, and phonophobia. These findings suggest that vaccine-related headaches can exhibit migraine-like features in some individuals [6]. In a separate online survey study involving 1372 fully vaccinated individuals, headache associated with COVID-19 vaccination was commonly described as a pressing sensation that involved the entire cranium. The majority of participants reported moderate-intensity headaches regardless of the vaccine type received. Additionally, the duration of these vaccine-related headaches was generally short, with a median of 2 days from onset to resolution [17]. In another observational study that utilized online questionnaires, 2464 individuals who received the AZD1222 (AstraZeneca) vaccine were assessed. The study found that headache typically began approximately 14.5 ± 21.6 h after vaccination and lasted for an average duration of 16.3 ± 30.4 h. The reported characteristics of the headache included bilateral location in 75.8% of participants, a pressing sensation in 50.4%, and a dull sensation in 37.7%. Regarding headache intensity, 38.7% of participants described it as severe, 35.2% as moderate, and 15.5% as very severe. Interestingly, this study also revealed that migraine-like symptoms, including aggravation of pain during physical activity and sensitivity to light and sound (photo/phonophobia), were reported by the participants who experienced headache after the vaccination. This suggests that the headache experienced in this context may share some similarities with migraine headaches, further highlighting the overlap in symptoms between COVID-19 vaccine-related headaches and migraine [18].

Although headache was mostly reported to be transient after vaccination, it sometimes can be resistant and long-lasting in some patients [14]. Especially in patients with a primary headache disorder, headache might be longer in duration and more severe/intense [1, 15]. According to the study conducted by Silvestro et al., using an online questionnaire, it was reported that among 841 migraine patients, 66.47% of patients after the 1^st^ dose and 60.15% of patients after the 2^nd^ dose of COVID-19 vaccination experienced a headache attack starting after 1 h to 7 days. These vaccine-related headaches were described as more severe (50.62%), long-lasting (52.80%), and longer in duration (49.69%) compared to their usual migraine attacks [19]. Sekiguchi et al. reported that the rates of COVID-vaccine-related headaches were reported as 30.9% after the 1^st^ dose and 66.2% after the 2^nd^ dose in patients with a primary headache history. In comparison, the rates were 13.6% after the 1^st^ dose and 32% after the 2^nd^ dose in individuals without a headache history. These findings suggest that patients with a pre-existing primary headache disorder may be more prone to experience vaccine-related headaches compared to those without a headache history [1]. The findings of our study are consistent with these previous studies mentioned. In patients with a pre-existing headache disorder who developed COVID-vaccination-related headaches, we observed a shorter onset of headache, with most headaches being described as throbbing in quality. Additionally, symptoms such as photophobia, phonophobia, and osmophobia were more prominent in these patients. These observations align with the notion that patients with a pre-existing headache disorder may experience distinct characteristics in their COVID-vaccination-related headaches.

In our study, gender was found to have no effect on the clustering of headache phenotypes (gender distribution was similar in both clusters; *p* = 0.550). Nonetheless, we believe that a prospective study with a larger sample of patients, specifically focusing on gender differences in terms of long COVID symptoms, including headache, would be beneficial for a more comprehensive understanding and management of headaches associated with COVID-19 vaccination.

Similarly, age had no significant effect on the formation of distinct headache clusters related to COVID-19 vaccination (*p* = 0.162, Supplementary table 4). Further prospective studies are required to draw more definitive conclusions on this matter.

As an additional noteworthy finding, the comparison of Mig-SCog scale scores between patient visits suggests that cognitive involvement in our group of patients with post-vaccination headache was not severe and exhibited a gradual improvement over time.

### Cluster analysis of headaches in patients with a prolonged/worsened COVID-19 vaccination-related headaches

There are only a few studies available that have examined the long-term course of prolonged/worsened COVID-19 vaccination-related headaches. In our study, we found that these headaches can be categorized into two distinct phenotypic clusters, and these phenotypes tend to maintain their characteristics over a 6-month follow-up period. The first cluster consisted of headaches that resembled migraine-like features, including throbbing pain, greater severity, and accompanying symptoms such as nausea, photophobia, and phonophobia. These headaches were exacerbated by physical activity and were more common in patients with a history of primary headaches. The second cluster included headaches with a tension-type phenotype, characterized by pressing or stabbing pain and longer duration (over 1 month). After 6 months of follow-up, 52.32% of patients in cluster 1 and 42.04% in cluster 2 still experienced ongoing (prolonged) headaches. Notably, patients in cluster 1 maintained their headache characteristics without significant changes over the 6-month period. Furthermore, we observed a decrease in headache severity across all patients throughout the 6 months of follow-up.

The exact pathophysiology underlying headaches related to COVID-19 vaccination is not yet fully understood, and several hypotheses are being explored. One hypothesis is related to the hyperexcitability of the trigeminovascular system (TVS) and the sensitization of trigeminal nerve fibers. It is believed that in patients with a pre-existing primary headache disorder, these mechanisms may lead to an increase in pain sensitivity and frequency [16, 19]. However, further research is needed to elucidate the precise mechanisms involved in COVID-19 vaccination-related headaches. Indeed, it is plausible that COVID-19 vaccination may stimulate the immune system [20], resulting in an immune response that could potentially trigger TVS. Activation of immune system cells can lead to the production of proinflammatory cytokines, including prostaglandins and bradykinins. These substances have the potential to induce the release of calcitonin gene-related peptide (CGRP) from neurons in the TVS, which is known to play a role in migraine headaches. This immune-mediated response may contribute to the development of migraine-like headaches following vaccination. However, further research is needed to establish a definitive link between immune system activation, TVS involvement, and vaccine-induced headaches [6, 16]. Also, inactivated COVID-19 vaccines, which often contain aluminum salt-based adjuvants, can modulate and induce an immune response. These adjuvants have the potential to stimulate the immune system and trigger the release of proinflammatory cytokines such as interleukin (IL)-1β and IL-18. These cytokines may play a role in triggering headache symptoms [6, 21, 22].

In this study, we provided evidence that patients with a pre-existing headache, particularly migraine, are more likely to exhibit the migraine-like headache phenotype in cluster 1, while the opposite group demonstrates characteristics resembling tension-type headaches. These findings support the notion of increased TVS hyperexcitability and heightened pain sensitivity in cluster 1. Our results suggest that individuals with a pre-existing migraine condition are more prone to experiencing persistent, resistant, and severe headaches following COVID-19 vaccination, which may require ongoing medical treatment. These patients may not experience headache remission after vaccination. As a result, it is crucial for headache specialists and clinicians to provide specialized care for these individuals in terms of headache management.

### Prolonged/worsened COVID-19 vaccination-related headaches as a result of other secondary etiologies

In our study, we encountered a relatively small number of cases (4.6%) with other secondary prolonged or worsened COVID-19 vaccination-related headaches, which limited our ability to draw definitive conclusions. However, it was observed that the majority of other secondary headaches were attributed to cerebral venous thrombosis, accounting for approximately 62.5% of the cases. This suggests that this specific cause may have a significant role in the development of serious secondary headaches following COVID-19 vaccination [3, 7].

Moreover, it is noteworthy that almost all patients (87.5%) with other secondary headache etiologies did not have a pre-existing primary headache condition. This emphasizes the importance of conducting a thorough examination in individuals experiencing new-onset headaches related to COVID-19 vaccination.

Further research with larger consecutive sample sizes is warranted to investigate and gain a better understanding of the association between COVID-19 vaccination and other secondary headache etiologies.

Vaccination-related headaches are not currently classified as a separate entity in the ICHD-3 classification system, despite their widespread recognition [12, 17]. Clinicians and researchers currently utilize the code "Headache attributed to the use of or exposure to a substance" (8.1) to categorize vaccination-related headaches. However, we believe that there is a need for a classification specifically for "COVID-19 vaccination-related headaches" in future versions of the ICHD classification system. This inclusion would be beneficial in recognizing the distinct phenotypes of these prolonged or worsened headaches and increasing awareness among clinicians responsible for differentiating and diagnosing these headaches in patients.

Although COVID-19 vaccination can lead to a variety of side-effects, it should be emphasized that “the long COVID phenomenon” itself is a much more serious healthcare problem globally in the long-term course after COVID-19 pandemic. People who recovered from acute COVID-19 might face a wide spectrum of persistent symptoms in the following weeks/months such as insomnia, fatigue, cognitive disturbance (brain fog), myalgia, hair loss, muscle weakness, psychiatric symptoms, and a persistent headache which creates a serious source of disability [23]. In our patients with a COVID-19 history, we also observed that fatigue (68.8%), cognitive impairment (45%) and hair loss (29.1%) was the three major long-COVID-19 symptoms. Hereby, vaccination against COVID-19 serves as a significant weapon against long-COVID-19 phenomenon, in order to prevent these clinical manifestations [24].

### Strengths and limitations

Our study possesses several notable strengths. Firstly, it is the first multinational and prospective study to investigate the long-term course of COVID-19 vaccination-related prolonged or worsened headaches. This provides valuable insights into the course of these headaches over time. Additionally, our study demonstrates that COVID-19 vaccination-related headaches exhibit distinct profiles and long-term courses. This highlights the existence of different phenotypes within these patient groups. The identification of these distinct phenotypes has the potential to influence treatment approaches for patients with COVID-19 vaccination-related headaches, allowing for more tailored and effective management strategies.

We acknowledge certain limitations in our study. Firstly, the inclusion of patients from diverse settings such as emergencies and outpatient policlinics introduces heterogeneity to our study group, which may affect the generalizability of our findings. Secondly, the non-consecutive nature of the patients included in the study may introduce selection bias, potentially leading to biased results. Conversely, patients with severe conditions might not be able to accurately express their headache symptoms in an outpatient setting, potentially affecting the completeness of the data. Lastly, Lastly, we were unable to analyze differences between various vaccine types due to the fact that over 75% of our study group had received at least one dose of an mRNA vaccine. Conducting further studies that specifically evaluate patients based on their COVID-19 vaccine types could yield more robust results for addressing this issue.

## Conclusions

Recognizing the two distinct phenotypes of COVID-19 vaccination-related prolonged or worsened headaches can have significant clinical benefits. These headaches can occur not only in patients with a pre-existing primary headache diagnosis but also in individuals who do not have any prior headache conditions. Being aware of the potential worsening of headaches after COVID-19 vaccination, especially in patients with a primary headache disorder like migraine, can serve as an early warning for clinicians and headache experts who are treating these patients. This awareness allows for preplanning and modification of treatment strategies to optimize patient care.

Furthermore, clinicians can proactively inform patients about the possibility of headache worsening or prolonged symptoms before administering COVID-19 vaccines. This can help manage patient expectations and potentially mitigate any anxiety or concerns related to the potential impact on their headaches.

### Supplementary Information

**Additional file 1.**

**Additional file 2.**

**Additional file 3.**

**Additional file 4.**

## Data Availability

All data and materials generated in this study are available upon request.

## Abbreviations

CGRP: Calcitonin gene related peptide
CT: Computerized tomography
f: Female
EMA: European Medicines Agency
GON: Great occipital nerve
ICHD: International Classification of Headache Disorders
IL: Interleukin
IHS: International Headache Society
MRI: Magnetic resonance imaging
m: Male
n: Number
%: Percentage
PH: Primary headache
SSRI: Selective serotonin reuptake inhibitors
SNRI: Serotonin noradrenaline reuptake inhibitor
SD: Standard deviation
TCADs: Tricyclic antidepressants
VRS: Verbal rating scale
TCADs: Tricyclic antidepressants
TVS: Trigeminovascular system

## References

[1] Sekiguchi K, Watanabe N, Miyazaki N (2022). Incidence of headache after COVID-19 vaccination in patients with history of headache: A cross-sectional study. Cephalalgia.

[2] - World Health Organization. Draft landscape of COVID-19 candidate vaccines https://www.who.int/publications/ m/item/draft-landscape-of-covid-19-candidate-vaccines (23 January 2023, accessed 02 February 2023.

[3] Garcia-Azorin D, Baykan B, Beghi E (2022). Timing of headache after COVID-19 vaccines and its association with cerebrovascular events: An analysis of 41,700 VAERS reports. Cephalalgia.

[4] Caronna E, van den Hoek TC, Bolay H (2023). Headache attributed to SARS-CoV-2 infection, vaccination and the impact on primary headache disorders of the COVID-19 pandemic: A comprehensive review. Cephalalgia.

[5] Kaur RJ, Dutta S, Bhardwaj P (2021). Adverse events reported from COVID-19 vaccine trials: A systematic review. Indian J Clin Biochem.

[6] Ekizoglu E, Gezegen H, Yalınay Dikmen P (2022). The characteristics of COVID-19 vaccine-related headache: Clues gathered from the healthcare personnel in the pandemic. Cephalalgia.

[7] García-Azorín D, Do TP, Gantenbein AR (2021). Delayed headache after COVID-19 vaccination: a red flag for vaccine induced cerebral venous thrombosis. J Headache Pain.

[8] - Vaccine Adverse Event Reporting System (VAERS). VAERS Data Sets. Available on: https://vaers.hhs.gov/data.html Accessed on February 2, 2023).

[9] Martelletti P (2023). COVID-19 and SARS-CoV-2 Vaccines: A Cameo Role for Headache. Int J Environ Res Public Health.

[10] Jensen MP, Wittink H, Carr D (2008). Pain assessment in clinical trials. Pain Management: Evidence, Outcomes and Quality of Life in Pain Treatment.

[11] Polat B, Özge A, Helvacı Yılmaz N (2020). Validity and reliability of the Turkish version of the mig-scog scale in migraine patients. Neurological Sciences and Neurophysiology.

[12] - Headache Classification Committee of the International Headache Society (IHS) (2018). The International Classification of Headache Disorders, 3rd edition. Cephalalgia 38: 1–211. 10.1177/0333102417738202 10.1177/0333102417738202 29368949

[13] - TIBCO Software Inc. (2018). Statistica (data analysis software system), version 13. http://tibco.com)

[14] Cocores AN, Goadsby PJ, Monteith TS (2023). Post-vaccination headache reporting: Trends according to the Vaccine Adverse Events Reporting System. Headache.

[15] Göbel CH, Heinze A, Karstedt S (2021). Clinical characteristics of headache after vaccination against COVID-19 (coronavirus SARS-CoV-2. with the BNT162b2 mRNA vaccine: a multicentre observational cohort study. Brain Commun.

[16] Ceccardi G, Schiano di Cola F, Di Cesare M (2022). Post COVID-19 vaccination headache: A clinical and epidemiological evaluation. Front Pain Res (Lausanne).

[17] Magdy R, Khedr D, Yacoub O (2022). Epidemiological aspects of headache after different types of COVID-19 vaccines: An online survey. Headache.

[18] Göbel CH, Heinze A, Karstedt S (2021). Headache attributed to vaccination against COVID-19 (Coronavirus SARS-CoV-2) with the ChAdOx1 nCoV-19 (AZD1222) vaccine: A multicenter observational cohort study. Pain Ther.

[19] Silvestro M, Tessitore A, Orologio I (2021). Headache Worsening after COVID-19 Vaccination: An Online Questionnaire-Based Study on 841 Patients with Migraine. J Clin Med.

[20] Naitlho A, Lahlou W, Bourial A (2021). A Rare Case of Henoch-Schönlein Purpura Following a COVID-19 Vaccine-Case Report. SN Compr Clin Med.

[21] Pulendran B, Ahmed R (2011). Immunological mechanisms of vaccination. Nat Immunol.

[22] Castaldo M, Waliszewska-Prosół M, Koutsokera M (2022). Headache onset after vaccination against SARS-CoV-2: a systematic literature review and meta-analysis. J Headache Pain.

[23] Martelletti P, Bentivegna E, Spuntarelli V (2021). Long-COVID Headache. SN Compr. Clin Med.

[24] Tana C, Bentivegna E, Cho SJ (2022). Long COVID headache. J Headache Pain.

