# Supplementary material for: COVID-19 vaccination-related headache showed two different clusters in the long-term course: a prospective multicenter follow-up study (COVA-Head Study)
Source: J Headache Pain. 2023 Sep 29;24(1):132. doi: 10.1186/s10194-023-01665-3 (PMC10541695; doi:10.1186/s10194-023-01665-3)
Supplement: Supplementary file 1 — Additional file 1. [file 10194_2023_1665_MOESM1_ESM.pdf]

Impact of COVID-19 Vaccination on Primary Headache Course (CoVaHead)

Dear investigator;
The frequency, clinical characteristics, phenotypes, and pathophysiological basis of headaches experienced following COVID-19 vaccines are a new area of investigation with numerous unanswered questions.

In this multicenter, multinational study, we aim to evaluate patients with COVID-19 vaccine-related headaches, who had been evaluated by experienced neurologists/headache experts like you.

It is of uttermost importance to remember that these questions should be answered by the physicians/neurologists with face-to-face interviews and not by the patient him/herself.

The patients will also be evaluated at baseline, 3rd month, and 6 th month on follow-up in order to follow the long-term course of the headaches.

Thank you for your valuable participation in our study.

---

*Required

1. Email *

____

2. Do you give consent to participate in this study? *

Mark only one oval.

- ( ) Yes
- ( ) No *Skip to section 22 (END OF QUERY)*

Patient's profile

3. Physician name for future contact *

____

4. Initials of the patient *

5. First admission date of the patient to the current neurology/headache center

Example: 7 January 2019

6. Date of enrollment *

Example: 7 January 2019

7. Date of birth *

Example: 7 January 2019

8. Gender of the patient *

Mark only one oval.

- ( ) Female
- ( ) Male

9. Did the patient have a primary headache diagnosis before? (Please check the diagnosis according to ICHD-3) *

Mark only one oval.

- ( ) Yes
- ( ) No *Skip to question 19*

Pre-existing primary headache diagnosis-1

10. Which primary headache disorder was the patient diagnosed with? (multiple selection) *

Tick all that apply.

- [ ] Migraine
- [ ] TTH (Tension type headache)
- [ ] TACs (Trigeminal autonomic cephalalgias)
- [ ] Medication overuse headache
- [ ] Other

11. The general frequency of the patient's previous primary headache *

Mark only one oval.

- ( ) less than 1 day per month *Skip to question 13*
- ( ) 1 day or more per month

headache days (pre-existing)

12. Headache days per month *

____

Pre-existing primary headache diagnosis-2

13. Quality of the previous primary headache ? (multiple selection) *

Tick all that apply.

- [ ] Throbbing
- [ ] Pressing
- [ ] Stabbing
- [ ] Other

14. Prominent location of the previous primary headache? (multiple selection) *

Tick all that apply.

- [ ] Frontal
- [ ] Vertex
- [ ] Temporal
- [ ] Occipital
- [ ] Holocranial
- [ ] Other

15. The side of the previous primary headache? *

Mark only one oval.

- ( ) Unilateral
- ( ) Bilateral
- ( ) Bilateral dominant on one side
- ( ) Unilateral but switch sides

16. Mean severity of the previous primary headache attacks *

Mild (1) non-irritating and did not interfere with my daily work; Moderate (2) uncomfortable but I was able to do my daily work; Severe (3) I couldn't do my daily work

Mark only one oval.

	1	2	3	
none	( )	( )	( )	severe

17. Please specify previous primary headache-related accompanying symptoms of the patient? (multiple selection) *

Tick all that apply.

- [ ] Nausea/vomiting
- [ ] Phonophobia
- [ ] Photophobia
- [ ] Osmophobia
- [ ] Diziness/vertigo
- [ ] Allodynia
- [ ] Cranial autonomic features
- [ ] Increase by physical activity
- [ ] Anosmia
- [ ] Ageusia
- [ ] Other

18. Does the patient currently use a preventive medicine for this primary headache disorder? *

Mark only one oval.

- ( ) Yes
- ( ) No

Comorbid medical condition

19. Does the patient have any comorbid medical condition? (multiple selection) *

Tick all that apply.

- [ ] Hypertension
- [ ] Diabetes mellitus
- [ ] Coronary artery disease
- [ ] COPD like chronic pulmonary diseases
- [ ] Asthma or other atopic disorders
- [ ] Epilepsy
- [ ] Stroke
- [ ] Neurodegenerative disorder
- [ ] Neuromuscular disorder
- [ ] Hypothyroidism
- [ ] Depression
- [ ] Anxiety disorder
- [ ] Hyperlipidemia
- [ ] Liver failure
- [ ] Renal disease
- [ ] Cancer
- [ ] Hyperthyroidism
- [ ] Rheumatological diseases
- [ ] Inflammatory bowel syndrome and/or primary chronic bowel problem
- [ ] Sleep problems/diseases
- [ ] Other
- [ ] None

20. Did the patient have a COVID-19 diagnosis? *

Mark only one oval.

- ( ) Yes
- ( ) No *Skip to question 40*

Covid-19 diagnosis

21. How was the clinical course of the COVID-19? *

Mark only one oval.

- Asymptomatic
- Symptomatic, at home but did not receive any specific treatment
- Treated at home
- Hospitalized in a regular hospital floor
- Hospitalized in ICU (intensive care unit)

22. How was the clinical involvement pattern? (multiple selection) *

Tick all that apply.

- [ ] Headache
- [ ] Neurological symptoms other than headache.
- [ ] Pulmonary involvement
- [ ] Other systemic conditions
- [ ] Other

23. Did the patient complain of ongoing fatigue after COVID-19 ? *

Mark only one oval.

- Yes
- No

24. Did the patient complain of cognitive blunting (brain fog) after the COVID-19? *

Mark only one oval.

- Yes
- No

25. Did the patient have a new diagnosis of a psychiatric illness (such as anxiety disorders, depression, ..etc) after COVID-19? *

Mark only one oval.

- Yes
- No

26. Did the patient complain of new onset sleep problems after COVID-19? *

Mark only one oval.

- Yes
- No

27. Did the patient complain of new onset hair loss after COVID-19? *

Mark only one oval.

- Yes
- No

28. Have the patient experienced a headache related to the COVID-19? *

Mark only one oval.

- Yes
- No *Skip to question 40*

COVID-19 related headache-1

29. When did headache start? Please specify the time relationship between headache and COVID-19 diagnosis ? *

Mark only one oval.

- ( ) Within 1 week before diagnosis
- ( ) At 0-71 hours following diagnosis
- ( ) After 72 hours-6 days following diagnosis
- ( ) After 1 week to 1 month following diagnosis
- ( ) After more than 1 month following diagnosis

30. How many days did the patient's headache last? *

____

31. Lateralization of headache during COVID-19? *

Mark only one oval.

- ( ) Unilateral
- ( ) Bilateral
- ( ) Bilateral dominant on one side
- ( ) Unilateral but switch sides

32. Quality of headache during COVID-19? (multiple choice is possible) *

Tick all that apply.

- [ ] Throbbing
- [ ] Pressing
- [ ] Stabbing
- [ ] Other

33. If the headache continued, what was the frequency of headache days per month? *

Mark only one oval.

- ( ) less than 1 day per month *Skip to question 35*
- ( ) 1 day or more per month

headache days (Covid related)

34. Headache days per month *

____

COVID-19 related headache-2

35. Prominent localization of headache? (multiple selection) *

Tick all that apply.

- [ ] Frontal
- [ ] Vertex
- [ ] Temporal
- [ ] Occipital
- [ ] Holocranial
- [ ] Other

36. Mean severity of the headache attacks related to COVID-19 *

Mild (1) non-irritating and did not interfere with my daily work; Moderate (2) uncomfortable but I was able to do my daily work; Severe (3) I couldn't do my daily work

Mark only one oval.

	1	2	3	
mild	( )	( )	( )	severe

37. Please specify COVID-19 headache-related accompanying symptoms of the patient? (multiple selection) *

Tick all that apply.

- [ ] Nausea/vomiting
- [ ] Photophobia
- [ ] Phonophobia
- [ ] Osmophobia
- [ ] Diziness/vertigo
- [ ] Allodynia
- [ ] Cranial autonomic features
- [ ] Increase by physical activity
- [ ] Anosmia
- [ ] Ageusia
- [ ] Other

38. How was the course of COVID-19 headache ? *

Mark only one oval.

- ( ) Improved without medication
- ( ) Totally relieved with medication
- ( ) Continued despite medication (no emergency referral)
- ( ) The patient had to be admitted to the emergency room/hospital because of headache

39. What is your clinical opinion as a physician about the relationship between the severity of COVID-19 and the severity of headache? *

Mark only one oval.

- ( ) Not correlated
- ( ) Correlated

COVID-19 vaccine related headache-1

40. Please specify patients' vaccination status

Tick all that apply.

	1 dose	2 doses	3 doses	4 doses
Pfizer-BioNTech	☐	☐	☐	☐
Sinovac (Coronavac)	☐	☐	☐	☐
Astra Zeneca	☐	☐	☐	☐
Moderna	☐	☐	☐	☐
Sputnik V	☐	☐	☐	☐
Johnson & Johnson’s Janssen	☐	☐	☐	☐
Sinopharm COVID-19 vaccine	☐	☐	☐	☐
Other vaccine	☐	☐	☐	☐

41. Did your patient experience headaches within 15 days after vaccination? *

Mark only one oval.

- Yes
- No *Skip to section 22 (END OF QUERY)*

COVID-19 vaccine related headache-2

42. After which dose/doses has she/he experienced headache? (multiple selection) *

Tick all that apply.

- ☐ After 1st dose
- ☐ After 2 nd dose
- ☐ After 3 rd dose
- ☐ After 4th dose

43. What was the vaccination date of the patient (Please indicate the date of the vaccination that was related with the longest headache that effects the patients' quality of life)

Example: 7 January 2019

44. How many days after did the "index" vaccine-related headache begin? (time relationship between vaccination that caused COVID-19 vaccine-related headache with the longest duration (index vaccine-related headache) (please enter the number of days from 0 to 15 between the vaccination and the begining of headache) *

45. How long did the headache last? *

Mark only one oval.

- ( ) Less than one day (in hours)
- ( ) 1 day or more *Skip to question 47*

Headache in hours

46. Tha duration of index headache in hours *

Skip to question 48

Headache in days

47. The duration of index headache in days *

COVID-19 vaccine related headache-3

48. The lateralization of headache during COVID-19 vaccination? *

Mark only one oval.

- Unilateral
- Bilateral
- Bilateral dominant at one side
- Unilateral but switch sides

49. Quality of headache? (multiple selection) *

Tick all that apply.

- [ ] Throbbing
- [ ] Pressing
- [ ] Stabbing
- [ ] Other

50. Did the headache last more than one month? *

Mark only one oval.

- Yes
- No *Skip to question 54*

51. Was the headache continuous without any remission period? *

Mark only one oval.

- Continuous *Skip to question 54*
- With remission

COVID-19 vaccine related headache frequency-1

52. Please give the frequency of headaches per month. *

Mark only one oval.

- less than 1 day per month *Skip to question 54*
- 1 day or more per month

COVID-19 vaccine related headache frequency-2

53. Headache days per month *

COVID-19 vaccine related headache-4

54. Prominent localization of headache? (multiple selection) *

Tick all that apply.

- [ ] Frontal
- [ ] Vertex
- [ ] Temporal
- [ ] Occipital
- [ ] Holocranial
- [ ] Other

55. Mean severity of the Covid-19 vaccine-related headache attacks *

Mild (1) non-irritating and did not interfere with my daily work; Moderate (2) uncomfortable but I was able to do my daily work; Severe (3) I couldn't do my daily work

Mark only one oval.

	1	2	3	
none	◯	◯	◯	severe

56. Please specify headache-related symptoms of the patient? (accompanying symptoms) (multiple choice is possible) *

Tick all that apply.

- [ ] Nausea/vomiting
- [ ] Phonophobia
- [ ] Photophobia
- [ ] Osmophobia
- [ ] Diziness/vertigo
- [ ] Allodynia
- [ ] Cranial autonomic features
- [ ] Increase by physical activity
- [ ] Anosmia
- [ ] Ageusia
- [ ] Other

57. How did the patient's headache improve? *

Mark only one oval.

- ( ) Improved without medication
- ( ) Totally relieved with medication
- ( ) Continued despite medication (no emergency referral)
- ( ) She/he had to went to the emergency room/hospital because of headache

58. Which medications/interventions were prescribed / applied to the patient to prevent or relieve the pain? (multiple selection) *

Tick all that apply.

- [ ] Paracetamol containing medicines (Parol, Minoset, Geralgine...)
- [ ] Anti-rheumatic painkillers (Apranax, Majezik, Arveles, Brufen, Advil, Etol...)
- [ ] Aspirin
- [ ] Ergotamine (Avmigran, Cafergot, Ergafein)
- [ ] Triptan (Migrex, Relpax, Imigran)
- [ ] Muscle relaxants (Muscoril, Sirdalud and similar)
- [ ] Botilinium toxin Type A injection
- [ ] Tricyclic antidepressants (TCAD)
- [ ] Selective seratonin reuptake inhibitors (SSRIs)
- [ ] Beta-blockers
- [ ] Selective noradrenaline reuptake inhibitors (SNRIs)
- [ ] Great occipital nerve blockage (GON)
- [ ] Gepants
- [ ] CGRP antibodies
- [ ] Steroids
- [ ] Other

59. Was the patient diagnosed with a secondary headache disorder in relation to COVID-19 vaccination? *

Mark only one oval.

- ( ) Yes
- ( ) No *Skip to question 72*

Secondary headache etiology

60. What was the etiology of this secondary headache? *

Mark only one oval.

- Cerebral venous trombosis
- Intracranial hypertension
- Meningoencephalitis
- Stroke
- Other

COVID-19 vaccine related headache-5

61. Which diagnostic tools did you have to use for the diagnostic evaluation of this secondary headache? (multiple selection) *

Tick all that apply.

- [ ] CT (cerebral tomography)
- [ ] CT angiography
- [ ] MRI (magnetic resonance imaging)
- [ ] MR angigography
- [ ] Angiography (DSA, ...etc)
- [ ] Cerebrospinal fluid analysis (CSF)
- [ ] Other

62. Was the patient's neuroimaging results normal or pathological? *

Mark only one oval.

- Normal
- Pathological

63. How was the patient's platelet count in routine complete blood count (CBC) examination at the time of the diagnosis of secondary headache? *

Mark only one oval.

- Decreased platelet count
- Increased platelet count
- Normal

64. Please indicate the platelet count of the patient at the diagnosis (in thousands), if available

___

65. How was the patient's D-dimer levels at the diagnosis? *

Mark only one oval.

- Increased D-dimer
- Decreased D-dimer
- Normal
- Not available

66. Please indicate the D-dimer level of the patient at the diagnosis, if available.

___

67. Please indicate the C-reactive protein (CRP) level of the patient at the diagnosis, if available.

___

68. Please indicate the sedimentation level of the patient at the diagnosis, if available? (exact value, in real numbers)

___

69. If performed, were the patient's CSF results normal or pathological? *

Mark only one oval.

- Normal
- Pathologic
- not known
- not done

70. How was the neurological examination of the patient? *

Mark only one oval.

- Normal
- Abnormal

71. Has the patient experienced any other side effects besides headaches after the COVID-19 vaccination (multiple selection)? *

Tick all that apply.

- [ ] Fatigue
- [ ] Muscle pain
- [ ] Joint pain
- [ ] Fever (over 38 degrees)
- [ ] Mild fever (38 degrees or below)
- [ ] Itching
- [ ] Shortness of breath
- [ ] Nausea / vomiting
- [ ] None

The Mig-SCog scale (Please apply this scale to your patient if he/she has a COVID-19 vaccination related headache)

Please answer these questions considering the vaccine-related headache.

This is a specific instrument to measure and assess subjective cognitive symptoms during migraine attacks. It is a Likert-type scale consisting of 9 items scored between 0 and 18. Questions (1-3) relate to theareas of attention/processing speed/orientation; questions 4- 5 correspond to planning/attention; 6 and 7 to language, and 8 and 9 refer to language: naming. Eventually, Mig-SCog investigates the areas that are causing the most complaints in patients during an attack, namely, executive functions (attention, planning, and orientation) and language (naming and language). The scoring of the answers is as follows; Often (2 points), Sometimes (1 point) and None (0 points). The total score is the sum of these scores. A high Mig-SCog score indicates a high frequency of cognitive symptoms .For our Turkish collagues; the validity and reliability of the Turkish version of the Mig-Scog Scale was published by Polat B et al, at 8/5/2020 in Neurological Sciences and Neurophysiology Journal.

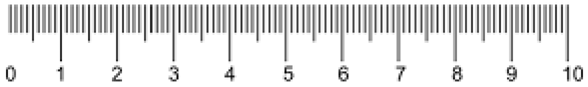

During your headaches;

72. Do you feel confused? *

Mark only one oval.

- Often
- Sometimes
- No

73. Do you have trouble performing tasks at your normal speed? *

Mark only one oval.

- Often
- Sometimes
- No

74. Do you have trouble following a route (by driving or walking)? *

Mark only one oval.

- Often
- Sometimes
- No

75. Do you have trouble thinking? *

Mark only one oval.

- Often
- Sometimes
- No

76. Do you have trouble maintaining the thread of your thoughts?? *

Mark only one oval.

- Often
- Sometimes
- No

77. Do you have trouble in understanding when being spoken to? *

Mark only one oval.

- Often
- Sometimes
- No

78. Do you have difficulty organizing a sentence or a conversation? *

Mark only one oval.

- Often
- Sometimes
- No

79. Do you have trouble speaking other people's names? *

Mark only one oval.

- Often
- Sometimes
- No

80. Do you have trouble remembering the correct names of objects? *

Mark only one oval.

- Often
- Sometimes
- No

END OF QUERY

THANK YOU FOR YOUR ATTENDANCE

This content is neither created nor endorsed by Google.

Google Forms
