# Supplementary material for: COVID-19 vaccination-related headache showed two different clusters in the long-term course: a prospective multicenter follow-up study (COVA-Head Study)
Source: J Headache Pain. 2023 Sep 29;24(1):132. doi: 10.1186/s10194-023-01665-3 (PMC10541695; doi:10.1186/s10194-023-01665-3)
Supplement: Supplementary file 3 — Additional file 3. [file 10194_2023_1665_MOESM3_ESM.pdf]

Impact of COVID-19 Vaccination on Primary Headache Course (CoVaHead) (sixth month visit form)

*Required

1. Email *

____

2. Physician name for future contact *

____

3. Initials of the patient *

____

4. Date of birth *

____
Example: 7 January 2019

5. Date of visit *

____
Example: 7 January 2019

6. Does the patient's headache continue? *

Mark only one oval.

( ) Yes *Skip to question 7*
( ) No *Skip to question 22*

Headache characteristics at the follow-up visit

7. What is the frequency of headache days per month? *

Mark only one oval.

- ⬭ less than 1 day per month *Skip to question 9*
- ⬭ 1 day or more per month *Skip to question 8*

headache (1)

8. Average headache days per month *

____

headache (2)

9. Average duration of headache *

Mark only one oval.

- ⬭ less than 24 hours *Skip to question 10*
- ⬭ 1 day or more *Skip to question 11*

headache (3)

10. Average duration of headache in hours *

____

headache (4)

11. Average duration of headache in days *

____

headache (5)

12. Quality of headache ? (multiple selection) *

Tick all that apply.

- [ ] Pressing
- [ ] Throbbing
- [ ] Stabbing
- [ ] Other

13. The side of the headache? *

Mark only one oval.

- ( ) Unilateral
- ( ) Bilateral
- ( ) Bilateral dominant on one side
- ( ) Unilateral but switch sides

14. Prominent localization of headache? (multiple selection) *

Tick all that apply.

- [ ] Frontal
- [ ] Vertex
- [ ] Temporal
- [ ] Occipital
- [ ] Holocranial
- [ ] Other

15. Please specify headache-related accompanying symptoms of the patient? (multiple selection) *

Tick all that apply.

- [ ] Nausea/vomiting
- [ ] Phonophobia
- [ ] Photophobia
- [ ] Osmophobia
- [ ] Diziness/vertigo
- [ ] Allodynia
- [ ] Cranial autonomic features
- [ ] Increase by physical activity
- [ ] Anosmia
- [ ] Ageusia
- [ ] Other

16. Mean severity of the headache attacks *

Mild (1) non-irritating and did not interfere with my daily work; Moderate (2) uncomfortable but I wasable to do my daily work; Severe (3) I couldn't do my daily work

Mark only one oval.

	1	2	3	
mild	◯	◯	◯	severe

17. Did the headache improve or worsen since the first visit? *

Mark only one oval.

◯ Improved *Skip to question 18*

◯ Worsened *Skip to question 19*

◯ Did not change *Skip to question 20*

Improved headache-1

18. If the headache improved, how much did it improve? *

Mark only one oval.

- 25% or less
- >25 to 50%
- >50 to 75%
- >75 to 100%

Skip to question 20

Worsened headache-1

19. How much did the headache worsen? *

Mark only one oval.

- 50% and/or less
- more than 50%

Preventive treatment-1

20. Did the patient use any preventive medicine to relieve the pain? *

Mark only one oval.

- No *Skip to question 22*
- Yes *Skip to question 21*

Preventive treatment-2

21. Which medications/interventions were prescribed / applied to the patient to prevent or relieve the pain? (multiple selection) *

Tick all that apply.

- [ ] Paracetamol containing medicines (Parol, Minoset, Geralgine...)
- [ ] Anti-rheumatic painkillers (Apranax, Majezik, Arveles, Brufen, Advil, Etol...)
- [ ] Aspirin
- [ ] Ergotamine (Avmigran, Cafergot, Ergafein)
- [ ] Triptan (Migrex, Relpax, Imigran)
- [ ] Muscle relaxants (Muscoril, Sirdalud and similar)
- [ ] Botilinium toxin Type A injection
- [ ] Tricyclic antidepressants (TCAD)
- [ ] Selective seratonin reuptake inhibitors (SSRIs)
- [ ] Beta-blockers
- [ ] Selective noradrenaline reuptake inhibitors (SNRIs)
- [ ] Great occipital nerve blockage (GON)
- [ ] Gepants
- [ ] CGRP antibodies
- [ ] Steroids
- [ ] Other

COVID-19

22. Did the patient have COVID-19 within the time period between the previous and current visits? *

Mark only one oval.

- ( ) Yes
- ( ) No

Long COVID questions

Please do not answer these questions in this section and skip to section 14 (Mig-SCog Scale) if the patient never had COVİD-19

23. When did the patient have COVID-19 diagnosis?

____

Example: 7 January 2019

24. Does the patient complain of ongoing fatigue after COVID-19 ?

Mark only one oval.

- Yes
- No

25. Does the patient have a new diagnosis of a psychiatric illness (such as anxiety disorders, depression, ..etc) after COVID-19 ?

Mark only one oval.

- Yes
- No

26. Does the patient complain of new onset sleep problems after COVID-19 ?

Mark only one oval.

- Yes
- No

27. Does the patient complain of new onset hair loss after COVID-19?

Mark only one oval.

- Yes
- No

The Mig-SCog scale

Please answer these questions considering the vaccine-related headache.

During your headaches;

28. Do you feel confused? *

Mark only one oval.

- Often
- Sometimes
- No

29. Do you have trouble performing tasks at your normal speed? *

Mark only one oval.

- Often
- Sometimes
- No

30. Do you have trouble following a route (by driving or walking)? *

Mark only one oval.

- Often
- Sometimes
- No

31. Do you have trouble thinking? *

Mark only one oval.

- Often
- Sometimes
- No

32. Do you have trouble maintaining the thread of your thoughts? *

Mark only one oval.

- Often
- Sometimes
- No

33. Do you have trouble in understanding when being spoken to? *

Mark only one oval.

- Often
- Sometimes
- No

34. Do you have difficulty organizing a sentence or a conversation? *

Mark only one oval.

- Often
- Sometimes
- No

35. Do you have trouble speaking other people's names? *

Mark only one oval.

- Often
- Sometimes
- No

36. Do you have trouble remembering the correct names of objects? *

Mark only one oval.

- Often
- Sometimes
- No

Additional Vaccination (1)

37. Did the patient have another vaccination of any kind (COVID, influenza, etc) between the previous and the current visits?

Mark only one oval.

- Yes
- No *Skip to section 17 (END OF QUERY)*

Additional Vaccination (2)

38. Please give the name of the vaccine applied to the patient *

____

39. Date of the vaccination?

____

Example: 7 January 2019

END OF QUERY

This content is neither created nor endorsed by Google.

Google Forms
