# Supplementary material for: COVID-19 vaccination-related headache showed two different clusters in the long-term course: a prospective multicenter follow-up study (COVA-Head Study)
Source: J Headache Pain. 2023 Sep 29;24(1):132. doi: 10.1186/s10194-023-01665-3 (PMC10541695; doi:10.1186/s10194-023-01665-3)
Supplement: Supplementary file 4 — Additional file 4. [file 10194_2023_1665_MOESM4_ESM.docx]

**Supplementary Table 4 a, b, c. Factors effecting clustering of patients**

1. **First Visit**

| First Visit | Cluster1  (n=86) | | Cluster2  (n=88) | | Total  (n=174) | | P value |
| --- | --- | --- | --- | --- | --- | --- | --- |
| Gender (Female) (n, %) | 65 | 75.58% | 63 | 71.59% | 128 | 73.56% | 0.550441 |
| Primary Headache (n, %) | 63 | 73.26% | 44 | 50.00% | 107 | 61.49% | 0.001513 |
| Comorbidity (n, %) | 60 | 69.77% | 56 | 63.64% | 116 | 66.67% | 0.390697 |
| Unilateral (n, %) | 34 | 39.53% | 32 | 36.36% | 66 | 37.93% | 0.666428 |
| Headache lasting more than one month (n, %) | 30 | 34.88% | 50 | 56.82% | 80 | 45.98% | 0.003554 |
| \| Throbbing (n, %) \| \| --- \| | 68 | 79.07% | 25 | 28.41% | 93 | 53.45% | 0.000000 |
| \| Pressing (n, %) \| \| --- \| | 29 | 33.72% | 67 | 76.14% | 96 | 55.17% | 0.000000 |
| \| Stabbing (n, %) \| \| --- \| | 9 | 10.47% | 19 | 21.59% | 28 | 16.09% | 0.043726 |
| \| Frontal (n, %) \| \| --- \| | 63 | 73.26% | 31 | 35.23% | 94 | 54.02% | 0.000000 |
| \| Vertex (n, %) \| \| --- \| | 15 | 17.44% | 18 | 20.45% | 33 | 18.97% | 0.612045 |
| \| Temporal (n, %) \| \| --- \| | 32 | 37.21% | 32 | 36.36% | 64 | 36.78% | 0.907924 |
| \| Occipital (n, %) \| \| --- \| | 28 | 32.56% | 26 | 29.55% | 54 | 31.03% | 0.667568 |
| \| Holocranial (n, %) \| \| --- \| | 15 | 17.44% | 20 | 22.73% | 35 | 20.11% | 0.383798 |
| \| Nausea (n, %) \| \| --- \| | 53 | 61.63% | 20 | 22.73% | 73 | 41.95% | 0.000000 |
| \| Phonophobia (n, %) \| \| --- \| | 66 | 76.74% | 14 | 15.91% | 80 | 45.98% | 0.000000 |
| \| Photophobia (n, %) \| \| --- \| | 70 | 81.40% | 11 | 12.50% | 81 | 46.55% | 0.000000 |
| \| Osmophobia (n, %) \| \| --- \| | 23 | 26.74% | 3 | 3.41% | 26 | 14.94% | 0.000005 |
| \| Dizziness (n, %) \| \| --- \| | 25 | 29.07% | 23 | 26.14% | 48 | 27.59% | 0.665111 |
| \| Cranial autonomic (n, %) \| \| --- \| | 10 | 11.63% | 6 | 6.82% | 16 | 9.20% | 0.270226 |
| \| Increase by physical activity (n, %) \| \| --- \| | 47 | 54.65% | 21 | 23.86% | 68 | 39.08% | 0.000026 |
| \| Allodynia (n, %) \| \| --- \| | 23 | 26.74% | 21 | 23.86% | 44 | 25.29% | 0.662046 |
| Age (mean±standard deviation) | 43.73±12.94 | | 46.56±13.67 | | 45.17±13.36 | | 0.162141 |
| Headache onset time after vaccination (mean±standard deviation) | 3.03±3.82 | | 3.10±3.39 | | 3.06±3.59 | | 0.902151 |
| Severity (mean±standard deviation) | 2.77±0.42 | | 2.18±0.65 | | 2.47±0.62 | | 0.000000 |

1. **Second visit (3rd month visit)**

| Second Visit | Cluster1  (n=50) | | Cluster2  (n=56) | | Total  (n=106) | | P value |  |
| --- | --- | --- | --- | --- | --- | --- | --- | --- |
| \| Throbbing (n, %) \| \| --- \| | 42 | 84.00% | 20 | 35.71% | 62 | 58.49% | 0.000000 |  |
| \| Pressing (n, %) \| \| --- \| | 20 | 40.00% | 41 | 73.21% | 61 | 57.55% | 0.000494 |  |
| \| Stabbing (n, %) \| \| --- \| | 5 | 10.00% | 10 | 17.86% | 15 | 14.15% | 0.241782 |  |
| \| Frontal (n, %) \| \| --- \| | 33 | 66.00% | 14 | 25.00% | 47 | 44.34% | 0.000017 |  |
| \| Vertex (n, %) \| \| --- \| | 7 | 14.00% | 10 | 17.86% | 17 | 16.04% | 0.587965 |  |
| \| Temporal (n, %) \| \| --- \| | 32 | 64.00% | 21 | 37.50% | 53 | 50.00% | 0.006135 |  |
| \| Occipital (n, %) \| \| --- \| | 15 | 30.00% | 18 | 32.14% | 33 | 31.13% | 0.811916 |  |
| \| Holocranial (n, %) \| \| --- \| | 3 | 6.00% | 9 | 16.07% | 12 | 11.32% | 0.094283 | |
| \| Nausea (n, %) \| \| --- \| | 37 | 74.00% | 15 | 26.79% | 52 | 49.06% | 0.000001 | |
| \| Phonophobia (n, %) \| \| --- \| | 43 | 86.00% | 16 | 28.57% | 59 | 55.66% | 0.000000 | |
| \| Photophobia (n, %) \| \| --- \| | 46 | 92.00% | 13 | 23.21% | 59 | 55.66% | 0.000000 | |
| \| Osmophobia (n, %) \| \| --- \| | 21 | 42.00% | 3 | 5.36% | 24 | 22.64% | 0.000003 | |
| \| Dizziness (n, %) \| \| --- \| | 11 | 22.00% | 11 | 19.64% | 22 | 20.75% | 0.765283 | |
| \| Cranial autonomic (n, %) \| \| --- \| | 4 | 8.00% | 3 | 5.36% | 7 | 6.60% | 0.584569 | |
| \| Increase by physical activity (n, %) \| \| --- \| | 30 | 60.00% | 11 | 19.64% | 41 | 38.68% | 0.000015 | |
| \| Allodynia (n, %) \| \| --- \| | 12 | 24.00% | 12 | 21.43% | 24 | 22.64% | 0.752295 | |
| \| Average duration of headache in days (mean±standard deviation) \| \| --- \| | 8.38±10.09 | 4.30±6.48 | 6.23±8.58 | 0.013954 | | | | |
| \| Mean severity of the headache attacks (mean±standard deviation) \| \| --- \| | 2.44±0.58 | 1.77±0.69 | 2.08±0.72 | 0.000000 | | | | |

1. **Third visit (6th month visit)**

| Third Visit | Cluster1  (n=45) | | Cluster2  (n=37) | | Total  (n=82) | | P value |
| --- | --- | --- | --- | --- | --- | --- | --- |
| \| Throbbing (n, %) \| \| --- \| | 34 | 75.56% | 11 | 29.73% | 45 | 54.88% | 0.000024 |
| \| Pressing (n, %) \| \| --- \| | 21 | 46.67% | 30 | 81.08% | 51 | 62.20% | 0.001088 |
| \| Stabbing (n, %) \| \| --- \| | 5 | 11.11% | 4 | 10.81% | 9 | 10.98% | 0.965456 |
| \| Frontal (n, %) \| \| --- \| | 32 | 71.11% | 9 | 24.32% | 33 | 40.24% | 0.000017 |
| \| Vertex (n, %) \| \| --- \| | 6 | 13.33% | 6 | 16.22% | 12 | 14.63% | 0.713754 |
| \| Temporal (n, %) \| \| --- \| | 29 | 64.44% | 9 | 24.32% | 38 | 46.34% | 0.000225 |
| \| Occipital (n, %) \| \| --- \| | 12 | 26.67% | 17 | 45.95% | 29 | 35.37% | 0.069023 |
| \| Holocranial (n, %) \| \| --- \| | 4 | 8.89% | 6 | 16.22% | 10 | 12.20% | 0.313859 |
| \| Nausea (n, %) \| \| --- \| | 37 | 82.22% | 8 | 21.62% | 45 | 54.88% | 0.000000 |
| \| Phonophobia (n, %) \| \| --- \| | 37 | 82.22% | 8 | 21.62% | 45 | 54.88% | 0.000000 |
| \| Photophobia (n, %) \| \| --- \| | 41 | 91.11% | 7 | 18.92% | 48 | 58.54% | 0.000000 |
| \| Osmophobia (n, %) \| \| --- \| | 15 | 33.33% | 2 | 5.41% | 17 | 20.73% | 0.000985 |
| \| Dizziness (n, %) \| \| --- \| | 10 | 22.22% | 6 | 16.22% | 17 | 20.73% | 0.492285 |
| \| Cranial autonomic (n, %) \| \| --- \| | 2 | 4.44% | 1 | 2.70% | 3 | 3.66% | 0.672118 |
| \| Increase by physical activity (n, %) \| \| --- \| | 27 | 60.00% | 6 | 16.22% | 33 | 40.24% | 0.000034 |
| \| Allodynia (n, %) \| \| --- \| | 4 | 8.89% | 8 | 21.62% | 12 | 14.63% | 0.103889 |
| \| Average duration of headache in days (mean±standard deviation) \| \| --- \| | 6.04±8.10 | | 4.76±7.73 | | 5.46±7.91 | | 0.467011 |
| \| Mean severity of the headache attacks (mean±standard deviation) \| \| --- \| | 2.35±0.57 | | 1.73±0.73 | | 2.07±0.72 | | 0.000040 |
